# A retrospective study on the influence of inclination of cusp on implant marginal bone height in patients with periodontal disease

**DOI:** 10.2340/aos.v83.41226

**Published:** 2024-09-12

**Authors:** Runsheng Pei, Cong Xiao, Jian Chen, Hao Liu, Jinting Chen, Haixia Ge, Nana Cai, Yihua Wu, Yan Zhou

**Affiliations:** a Department of prosthodontics, The Affiliated Nantong Stomatological Hospital of Nantong University, Nantong, Jiangsu province, P.R. China; b Department of Orthodontics, The Affiliated Nantong Stomatological Hospital of Nantong University, Nantong, Jiangsu province, P.R. China; c The Affiliated Nantong Stomatological Hospital of Nantong University, Nantong, Jiangsu province, P.R. China; d Department of Periodontology, The Affiliated Nantong Stomatological Hospital of Nantong University, Nantong, Jiangsu province, P.R. China

**Keywords:** Inclination of cusp, periodontal disease, marginal bone height, occlusal design

## Abstract

**Purpose:**

To investigate the correlation between the marginal bone height of implants in the posterior maxilla of patients with periodontal disease and the inclination of cusp, providing a theoretical basis for the occlusal design of implant restorations in such patients.

**Methods:**

A total of 80 patients with periodontal disease who underwent implant restoration in the posterior maxilla (55 men and 25 women; mean age 56.66 ± 12.70 years) were selected, with a total of 80 implant restorations (one implant restoration per patient). In addition to recording the main research factor of the inclination of cusp, general patient information, implant characteristics and restoration characteristics were taken, and retrospective analysis of the case data and imaging data of the 80 patients from over 3 years was conducted. Cone beam computed tomography was performed preoperatively and 3 years after implant loading to measure and calculate the marginal bone height of the implants using the One Volume Viewer software. Correlation analysis was performed to determine the relationship between the inclination of the cusp and marginal bone height.

**Results:**

There was a positive correlation between the inclination of cusp and the marginal bone height of the implants, with a correlation coefficient of 0.661 (*p* < 0.001); the diameter of the implants, implant type and restoration type were negatively correlated with the marginal bone height of the implants, with correlation coefficients of −0.364 (*p* = 0.001), −0.232 (*p* = 0.038) and −0.298 (*p* = 0.007), respectively.

**Conclusion:**

When designing the occlusion of implant restorations in the posterior maxilla of patients with periodontal disease, it is advisable to appropriately reduce the restoration’s inclination of cusp.

## Introduction

The marginal bone height around implants is an important indicator of implant success and a major topic of research and discussion in the field of oral implantology [1, 2]. Pierluigi provided a descriptive review of the reasons and explanations for changes in marginal bone height around dental implants, suggesting that occlusal overload or biomechanical factors may contribute to peri-implant bone loss [3]. Melsen and Lang observed that in monkeys, bone deposition around implants occurred when micro-strain levels reached 3,400–6,600 [4–6], and net bone loss occurred after reaching a threshold of 6,700 micro-strains [7, 8]. In recent years, an increasing amount of literature has emphasized the importance of understanding and managing the response of implants to occlusal forces. While the occlusion and trauma of natural teeth have been extensively studied, literature on occlusion of implant restorations is limited [9–12]. The biological and physical differences between teeth and implants make it almost impossible to apply occlusal principles for natural teeth to implant-supported dental implants. Furthermore, studying implant occlusion poses several challenges, including feasibility and ethical issues in human clinical research [13, 14]. Therefore, much of the available information on implant occlusion relies on engineering and mechanical principles to understand implant occlusion, with very few clinical studies available. This study aims to investigate the correlation between the marginal bone height of implants in the posterior maxilla of patients with periodontal disease and the inclination of the cusp. This research seeks to provide a theoretical basis for the occlusal design of implant restorations in such patients, addressing a critical gap in current clinical knowledge and practice.

## Materials and methods

### Patient demographics

This study enrolled patients diagnosed with periodontal disease requiring implant-supported dental restorations in the posterior maxillary region, and the patients’ consent and voluntary participation were obtained. The participants were treated at the implantology and prosthodontics department of Nantong Stomatological Hospital between January 2016 and December 2020. A total of 80 patients, constituting 80 dental implants, were included in the research cohort. The data collected encompassed patient demographics, such as gender and age, as well as implant-related variables, including the implant system, length, diameter and marginal bone height around the implant. Additionally, parameters concerning the implant restoration, such as the inclination of cusp and crown-to-implant ratio, were recorded. The type of restoration and marginal bone height around the adjacent natural tooth were also documented.

The inclusion criteria were as follows: (1) voluntary acceptance of implant restoration for missing premolars or molars in the maxilla, with preoperative cone beam computed tomography (CBCT) imaging and a minimum of 3 years of postoperative CBCT scans available; (2) diagnosis of periodontal disease by the hospital’s periodontal department before implant surgery, with effective disease management and the adjacent dentition being natural teeth; (3) favorable bone quality in the posterior maxillary region, absence of severe alveolar bone ridge resorption, and flat alveolar bone in the edentulous area; (4) uneventful surgical proceedings with no postoperative infections or complications; (5) good patient compliance, enabling timely completion of follow-up visits for the study; and (6) overall good general health. This study was conducted in accordance with the principles of the Declaration of Helsinki. Ethical approval was obtained from the ethics committee of Nantong Stomatological Hospital.

### Research methodology

Two implant systems, Straumann and Dentium, were included in this study. The standard implantation techniques recommended by each system were employed, utilizing a flap approach for implant placement with non-submerged healing. The CBCT images were captured preoperatively and at least 3 years post-loading. Measurements were performed using the measurement tools provided by the One Volume Viewer software.

#### Inclination of cusp measurement

The measurement and analysis of digital denture designs or postoperative digitised scans of STL data were conducted using the Surfacer software. The occlusal plane served as the reference plane for the measurements. In cases of implant displacement, the inclination of cusp was determined using the functional cusps with occlusal contacts [15].

#### Implant marginal bone height measurement

**Figure 1:**
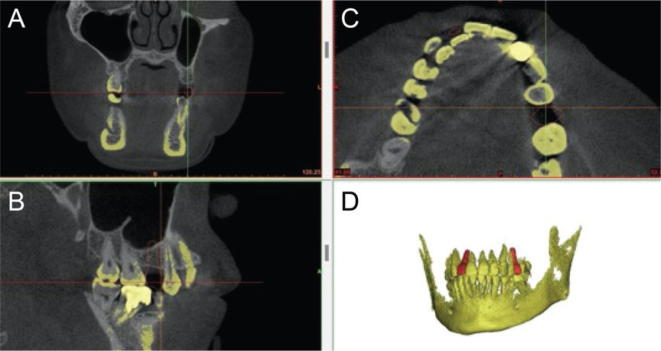
Alignment of CBCT images before implantation and 3 years after implant loading, with the yellow portion in the images representing CBCT images three years after implant loading. Figure 1A is a coronal section screenshot of the aligned images, Figure 1B is a horizontal section screenshot of the aligned images, Figure 1C is a sagittal section screenshot of the aligned images, and Figure 1D is a 3D screenshot of the aligned images.

**Figure 2:**
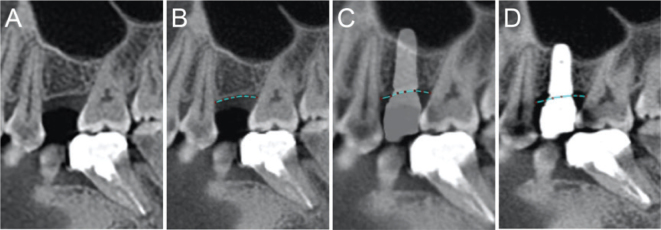
Comparison analysis of CBCT images before implantation and 3 years after implantation, with Figures 2A and 2B representing pre-implantation CBCT images, and Figures 2C and 2D representing CBCT images 3 years after implant loading. The blue dashed line in Figures 2B, 2C, and 2D indicates the alveolar bone margin before implantation.

The CBCT images taken before implant placement and 3 years after implant loading were superimposed (refer to Figure 1). The preoperative alveolar bone position was identified on the CBCT image captured 3 years after implant loading and marked (refer to Figure 2). Utilizing the length measurement tool in the One Volume Viewer software, the CBCT images were cropped in the mesial and distal directions passing through the center of the implant. At two points (mesial and distal to the implant, parallel to the long axis of the implant), the distance from the lowest point of the alveolar bone resorption area around the implant neck to the marked line was measured. The measurements at these two points were averaged.

#### Measurement of marginal bone height of adjacent natural teeth

Comparison and analysis of the CBCT images of adjacent natural teeth were conducted before implant placement and 3 years after implant loading. On the CBCT images taken 3 years after implant loading, the preoperative position of the marginal bone of the adjacent natural teeth was identified and marked. Utilizing the length measurement tool in the One Volume Viewer software, the CBCT images were cropped in the mesial and distal directions passing through the center of the adjacent natural teeth. At two points (mesial and distal to the adjacent natural teeth, parallel to the long axis of the tooth), the distance from the lowest point of the alveolar bone resorption area around the natural tooth neck to the marked line was measured. The measurements at these two points were averaged.

#### Crown/implant ratio of implant restorations

The height from the crown to the platform of the implant and the length of the implant were measured with reference to the data obtained when the patient wore the prosthesis. In cases where the data during prosthesis wearing were unavailable, the CBCT data taken 3 years after implant loading were used as a reference. The height from the crown to the platform of the implant and the length of the implant were measured using the length measurement tool in the One Volume Viewer software.

#### Observation indicators and correlation analysis

The primary observation indicator was the marginal bone height around the implants. Secondary indicators included the inclination of the cusp, implant diameter, implant type, restoration type, and the crown-to-implant ratio. To determine the relationships between the observed indicators, correlation analysis was conducted. The primary variables of interest included the inclination of the cusp, implant diameter, implant type, restoration type, crown-to-implant ratio, and the marginal bone height of implants.

### Statistical analysis

Due to the sample size (*N* = 80 > 50), the Kolmogorov–Smirnov test was used to assess normality. Only the implant marginal bone height followed a normal distribution (*p* = 0.200 > 0.05), while other indicators did not. For comparing the marginal bone height of implant restorations with different cusp inclinations, one-way analysis of variance (ANOVA) was employed. Spearman correlation coefficient was used for non-normally distributed variables such as the inclination of the cusp and the marginal bone height of adjacent natural teeth. Point-biserial correlation was used to analyze the relationship between binary categorical variables (gender, implant type, restoration type) and the continuous variable, implant marginal bone height.

## Results

### Patient demographics

Among the 80 samples, with 55 men (68.8%) and 25 women (31.2%), the mean age of the patients was 56.66 ± 12.70 years, with 26 patients (32.5%) under the age of 50 and 54 patients (67.5%) aged 50 or older. Single restorations accounted for 58.75%, and coronary prosthesis accounted for 41.25%. Among all these restorations, the inclination of the tooth apex is less than 15 °, accounting for 23.75%. The inclination of the dental apex between 15 ° and 25 ° accounts for 31.25%, respectively. The inclination of the tooth tip greater than 25 ° accounts for 45%. Patient demographics are presented in Table 1, and the grading and assignment of relevant factors are detailed in Table 2.

**Table 1:** General information of patients.

Item	Classification	Number (cases)	Proportion (*n*%)
Gender	Male	55	68.8%
	Female	25	31.2%
Age	56.66 ± 12.70		
	<50	26	32.5
	≥50	54	67.5
Inclination of cusp	<15°	19	23.75
	15°~25°	25	31.25
	>25°	36	45
Type of Implant	ITI	16	20
	Dentium	64	80
	Diameter 3.6 mm	6	7.5
	Diameter 4.0 mm	6	7.5
Diameter of implant	Diameter 4.1 mm	10	12.5
	Diameter 4.5 mm	19	23.75
	Diameter 4.8 mm	6	7.5
	Diameter 5.0 mm	33	41.25
Length of implant	8.0	17	21.25
	10.0	63	78.75
Type of restoration	Single	47	58.75
	Bridge	33	41.25
Crown-to-implant ratio	0.92 ± 0.26	-	-

**Table 2:** Classification of relevant factors.

Correlated factors	Variables	Negative value explanation
Gender	X_1_	Male = 1, Female = 2
Age	X_2_	< 50 = 1, ≥ 50 = 2
Inclination of cusp	X_3_	< 15° = 1
		15° ~ 25° = 2
		> 25° = 3
Type of implant	X_4_	ITI (Tissue-Level) = 1
		Dentium (Bone-Level) = 2
Diameter of implant	X_5_	Diameter 3.6 mm = 1
		Diameter 4.0 mm = 2
		Diameter 4.1 mm = 3
		Diameter 4.5 mm = 4
		Diameter 4.8 mm = 5
		Diameter 5.0 mm = 6
Length of implant	X_6_	8.0 = 1
		10.0 = 2
Type of restoration	X_7_	Single = 1
		Bridge = 2
Crown-to-implant ratio	X_8_	0.92 ± 0.26
Marginal bone height around the adjacent natural tooth (mm)	X_9_	0.69 ± 0.60
Marginal bone height around the implant (mm)	Y	1.96 ± 0.63

### Normality tests

The results of the normality tests for relevant factors are displayed in Table 3, indicating that only implant marginal bone height followed a normal distribution, whereas other factors did not.

**Table 3:** The normality test.

Item	Kolmogorov-Smirnov^a^			Shapiro-Wilk		
	Statistic	df	Sig.	Statistic	df	Sig.
Age	0.248	80	0.000	0.886	80	0.000
Gender	0.189	80	0.000	0.881	80	0.000
Inclination of cusp	0.160	80	0.000	0.931	80	0.000
Diameter of implant	0.238	80	0.000	0.828	80	0.000
Crown-to-implant ratio	0.151	80	0.000	0.917	80	0.000
Type of implant	0.108	80	0.022	0.960	80	0.013
Type of restoration	0.139	80	0.001	0.960	80	0.014
Marginal bone height around the adjacent natural tooth	0.148	80	0.000	0.854	80	0.000
Marginal bone height around the implant	0.080	80	0.200*	0.980	80	0.235

* This is the lower bound of the true significance level.

a Lilliefors Significance level correction.

### ANOVA results

The ANOVA results for the marginal bone height of implant restorations after 3 years of loading, with three different cusp inclinations, are shown in Table 4. The marginal bone height of implants with a cusp inclination of > 25° was 2.35 ± 0.56 mm, which was higher than in the other two groups (*p* < 0.001), demonstrating statistically significant differences.

**Table 4:** The effect of cusp inclination on peri-implant marginal bone height: A one-way analysis of variance (ANOVA).

Inclination of cusp	Marginal bone height around the implant (mm)	*p*-value
< 15°	1.41 ± 0.55	
15° ~ 25°	1.81 ± 0.39	< 0.001*
> 25°	2.35 ± 0.56	

Tukey HSD multiple comparisons: ‘< 15°’ versus ‘15° ~ 25°’ *p* = 0.036*; ‘< 15°’ versus ‘> 25°’ *p* < 0.001*; ‘15° ~ 25°’ versus ‘ > 25°’ *p* < 0.001*.

### Correlation analysis

The results of correlation analysis between the inclination of cusp and other factors with the marginal bone height of implant restorations after 3 years of loading are presented in Table 5. The inclination of cusp showed a positive correlation with the marginal bone height of implant restorations after 3 years of loading, with a correlation coefficient of 0.661 (*p* < 0.001). Conversely, the implant diameter, implant type and restoration type exhibited negative correlations with the marginal bone height of implant restorations after 3 years of loading, with correlation coefficients of −0.364 (*p* = 0.001), −0.232 (*p* = 0.038) and −0.298 (*p* = 0.007), respectively.

**Table 5:** Correlation analysis of factors with peri-implant cervical marginal bone height.

	Factors	Correlation coefficient	Statistic	p-value
Marginal bone height around the implant	Inclination of cusp	0.661	Spearman	<0.001*
	Marginal bone height around the adjacent natural tooth	−0.007	Spearman	0.950
	Gender	−0.054	Point-biserial	0.635
	Age	−0.050	Point-biserial	0.662
	Type of implant	−0.232	Point-biserial	0.038*
	Diameter of implant	−0.364	Spearman	0.001*
	Length of implant	0.075	Spearman	0.510
	Type of restoration	−0.298	Point-biserial	0.007*
	Crown-to-implant ratio	0.003	Spearman	0.976

* According to the significance level of α = 0.05, the differences are statistically significant.

## Discussion

With tooth loss and alveolar bone remodeling, buccal and lingual bone plates tend to resorb inwardly, causing implants to be placed more buccally or lingually than ideal, leading to off-axis biting loads on implant crowns. Rachel A. Sheridan noted that the inclination of the cusp in implant restorations is closely associated with occlusal overload [16]. Finite element analysis studies have shown that a higher cusp inclination increases stress on implant restorations and their supporting bone. Weinberg reported that a 10° increase in cusp inclination raises occlusal loads by an average of 30% [17]. Furthermore, a 30° cusp inclination subjected to a 2-mm off-axis force results in higher bone remodeling rates and greater bone-implant interface stress [18, 19]. These findings suggest that reducing cusp inclination may decrease bone strain and enhance implant stability. However, in-vitro experiments cannot fully simulate the complex biting environment and biological responses of the alveolar bone. Ethical and measurement limitations make clinical studies challenging; thus, a retrospective analysis of a smaller sample of patients with periodontal disease was chosen for this experiment.

Jiang BQ et al. indicated that patients with periodontitis undergoing implant treatment may exhibit different marginal bone absorption outcomes compared with periodontally healthy individuals [20]. The consensus regarding peri-implant bone loss suggests that marginal bone height decreases by approximately 1.6 mm within 3 years of implantation. In this study, when the inclination of cusp was <25°, the marginal bone height of implants after 3 years of loading was 1.63 ± 0.50 mm; in the case of the inclination of cusp exceeding 25°, the marginal bone height after 3 years was 2.35 ± 0.56 mm, indicating greater marginal bone height in implants with a higher inclination of cusp. The marginal bone height around adjacent natural teeth of implant restorations in this study was 0.69 ± 0.60 mm after 3 years, which is comparable to the physiological rate of alveolar bone resorption of 0.2 mm per year in non-periodontal diseased natural teeth, suggesting good periodontal disease control among the patients included in this study. Additionally, according to Klokkevold and Han (2007), effectively controlled periodontitis seems to have a limited impact on the survival rate of implants [21]. However, in this study, implants with an inclination of cusp exceeding 25° exhibited higher marginal bone height after 3 years (2.35 ± 0.56 mm).

According to the study by Yuye Cheng et al. on the effect of the inclination of cusp on marginal height of implant restorations under normal periodontal conditions, when restoration design featured a high inclination of cusp (>25°), the amount of alveolar bone resorption around implant restoration neck was 1.09 ± 0.23 mm [22], indicating a potentially slight pronounced effect of the inclination of cusp on the marginal bone height of implant restorations in patients with periodontal disease. The statistical results of the correlation analysis revealed a correlation coefficient of 0.554 between the inclination of cusp and implant marginal bone height, demonstrating a positive correlation. These findings collectively suggest a more significant impact of the inclination of cusp on the marginal bone height of implant restorations in patients with periodontal disease.

Kozlovsky’s 2007 experiment included inflammation and the occlusal high point as factors affecting implant marginal bone height over a relatively long period (12 months) [23]. The results indicated that inflammation around implants exacerbated bone resorption caused by plaque accumulation under occlusal overload. In the absence of inflammation around implants, slight marginal bone resorption occurred due to implant overload, but it did not exceed the implant neck.

Regarding implant diameter, length and crown-to-root ratio, most studies suggest that these factors have no significant impact on implant success rates. As for the crown-to-root ratio of implant restorations, the recent consensus from the European Association for Osseointegration states that a crown-to-root ratio not exceeding 2.2 does not affect the likelihood of biological complications associated with implant restorations, nor does it significantly affect marginal bone loss [24]. In this study, the crown-to-root ratio was 0.92 ± 0.26, which did not exceed this range, possibly contributing significantly to the results of this experiment.

Regarding implant length, most scholars believe that it has no significant effect on alveolar bone absorption around the implant neck within a certain range. However, Moriwaki’s study suggested that implants sized 5 × 6 mm exhibited better stress distribution than those sized 4 × 13 mm [25]. The consensus proposed by the ITI in 2018 suggests that an implant diameter of >4 mm ensures implant success rates; the current literature suggests that short implant lengths fall within the range of <6 mm. Additionally, the results of this experiment show a negative correlation coefficient of −0.364 between implant diameter and implant marginal bone height. Studies indicate that larger implant diameters result in less marginal bone resorption around the implant neck [26–28]. However, thicker implants are not always better, as excessively thick implants can lead to inadequate thickness of surrounding bone walls, resulting in marginal bone resorption around the implant neck.

In this study, implant type was negatively correlated with alveolar bone resorption around the neck of implants in patients with periodontal disease. This may be due to the selection of two types of implants for the experiment: bone-level implants and soft tissue-level implants from Dentium. Studies have shown that in posterior regions, bone-level implants exhibit less bone resorption around them compared with soft tissue-level implants [29–31]. This could be a significant factor contributing to the results of the experiment.

In this study, the restoration type was negatively correlated with alveolar bone resorption around the neck of implants in patients with periodontal disease. Most scholars believe that implant-supported crowns effectively distribute stress, reducing marginal bone resorption around the implant neck. The results of this experiment also confirm this point [32]. However, some systematic reviews and meta-analyses do not support the routine use of implant-supported crowns to prevent occlusal overload because the self-cleansing effect around the implant neck during crown restoration is poor, which can lead to biological complications [33]. It is worth further exploring which factor between stress dispersion and the self-cleansing effect around the implant neck plays a more critical role in preventing marginal bone resorption around the implant neck.

This study identified a significant positive correlation between cusp inclination and marginal bone height, suggesting that higher cusp inclinations result in increased marginal bone height. Implant diameter, type, and restoration type showed negative correlations with marginal bone height, providing insights into how these variables influence implant success. The findings suggest that reducing the cusp inclination in implant restorations for patients with periodontal disease can minimize bone stress and enhance implant stability. In addition, as a retrospective study, it relies on previously recorded data, which may limit the control over variables and data accuracy. The study’s sample size, although sufficient for initial findings, may not be large enough to generalize the results across all patient populations with periodontal disease. The study was conducted at a single institution, which may limit the generalizability of the findings to other settings or populations. The study only covers a three-year follow-up period, which may not be sufficient to observe long-term effects of cusp inclination on marginal bone height.

In summary, the impact of the inclination of cusp on the marginal bone height of dental implants in patients with periodontal disease is noteworthy. During implant restoration, patients with periodontal disease should actively consider the influence of occlusal factors on implants and appropriately reduce the inclination of cusp.
